# Constructing HLM to examine multi-level poverty-contributing factors of farmer households: Why and how?

**DOI:** 10.1371/journal.pone.0228032

**Published:** 2020-01-24

**Authors:** Yuewen Jiang, Chong Huang, Duoduo Yin, Chenxia Liang, Yanhui Wang

**Affiliations:** 1 3D Information Collection and Application Key Lab of Education Ministry, Capital Normal University, Beijing, China; 2 State Key Lab of Resources and Environmental Information System, Institute of Geographic Sciences and Natural Resources Research, Chinese Academy of Sciences, Beijing, China; Institute for Advanced Sustainability Studies, GERMANY

## Abstract

Accurately identifying poverty-contributing factors of farmer households in an all-round way is the critical prerequisite and guarantee for taking targeted measures in poverty alleviation. From the combined perspectives of multi-level comprehensive detection and human-nature sustainable development, this study has designed a multi-level index system of household-level, village-level, and town-level, and constructed a nested three-level hierarchical linear model to examine the poverty-contributing factors of farmer households, and to reveal the significant ones and their multi-level interaction mechanism. The case test from Fugong County shows that: (1) Poverty-contributing factors are multi-level, showing both individual and background effects. 77.14% of the poverty is caused by household-level factors, 6.24% by village-level ones and 16.62% by town-level factors. (2) Significant poverty-contributing factors at different levels are different, identifying different contribution degrees to poverty gaps of farmer households. Five household-level factors show significant influence on poverty degree and account for 70.95% of the overall poverty gap among poor households, 11.70% for four village-level significant factors and 86.80% for two town-level ones, respectively. (3) Higher-level factors have different degrees of influence on the contribution difference of lower-level ones. The two town-level factors, terrain relief and town per capita annual income have explained 59.38% of the difference of village-level proportion of migrant workers’ contribution to poverty degree among towns and 89.89% of the difference of household-level per capita annual income's contribution to poverty degree among towns respectively. (4) Measures such as improving the type of access to roads, developing characteristic planting and breeding, and implementing relocation projects, can help poor households in the study area to reduce poverty. This study provides a new perspective for identifying farmers' poverty-contributing factors and technical reference and decision support for local departments to plan and implement targeted assistance and household-specific development policies.

## Introduction

Poverty is a major global problem, and it is of vital importance for harmonious and sustainable development of the whole society. China is the largest developing country in the world and its poverty reduction plays a prominent role in achieving global poverty reduction goals [1, 2]. Among them, the poverty problem of farmers has always been the key consideration when the Chinese government makes a series of poverty alleviation policies [3]. Particularly since 2015, the Chinese government started to carry out an overall “precise poverty alleviation” strategy for breaking through the bottleneck of poverty reduction, which requires precise measures for each farm household and maintains the sustainable and coordinated development of society, economy, resources and environment [4]. As the basic livelihood unit in rural society, farmer households bear multiple risks resulting from environmental climate change and socioeconomic policies, and face multiple economic and social pressures due to changes in the surrounding ecological environment and socioeconomic development, which will undoubtedly increase the vulnerability of farmers’ livelihoods [5, 6]. In rural communities with limited income, access to education, medical care, and social security become the main social exclusion factors restricting the livelihood maintenance of farmers and threatening social welfare. Various activities, i.e., economic, social, and ecological, can contribute significantly to livelihood security [5, 7, 8]. Therefore, it is both critical and urgent to accurately detect poverty-contributing factors of rural poor households and further improve their ability to create sustainable livelihoods, which would provide reliable guidance and technical support for solving such poverty-related problems as "why the farmer households are poor?” and "how to help them”.

Exploring the causes of poverty has received considerable attention from scholars all over the world because of its universality, stubbornness and importance. Scholars in different fields tried to interpret and explore the problem by different methods [9–14]. A series of related studies from different perspectives (e.g., history, environment, capital shortage, cultural poverty, policy deficiencies, geographical location, ecological environment, family, individual factors and so on), show that the farmers’ poverty is affected not only by the low economic income, but also by more resource factors, such as natural condition, social development, and ecological environment, resulting from comprehensive interaction of multiple factors [1, 12, 15, 16]. The traditional methods of detecting poverty-contributing factors were mainly based on single-level statistical regression and spatial regression analysis considering spatial dependence, and had their own deficiencies. Specifically, popular regression methods, such as ordinary least squares (OLS), multiple linear regression and geographical weighting regression (GWR) [17–19], could only explore the single-level poverty-contributing factors that indicate individual effect (i.e., variation caused by the individual’s own characteristics), but couldn’t account for multi-level background effects (variation caused by the environment in which an individual lives, also known as environmental effect or pond effect). However, many studies show that [20–23], poverty-contributing factors of farmers are not only limited to the detection of a single population structure, economic development, and individual characteristics, but also affected by multi-level and multidimensional factors. In other words, Farmers' livelihood may be affected not only by their own individual characteristics and economic activities, but by surrounding ecological environment, socioeconomic development and other multi-dimensional background factors at the higher-level units where they live, especially the ecological environment and geographical location conditions have become important restrictive factors for the development of farmers, indicating the essential requirement of the harmonious and sustainable development of human and nature. The livelihood approach provides important knowledge for identifying key elements in farmers' daily living practices. The vulnerability approach provides a three-dimensional framework for analyzing livelihoods at risk because of external change [24]. Although there appears to be a growing recognition that merging livelihoods and vulnerability approaches may provide useful insights into questions of local-level livelihood, few studies have attempted to blend the livelihood framework with social exclusion and vulnerability from a multi-level perspective.

Obviously, in the case of increasing diversification of poverty-contributing factors, the scientific and reliable characters of the detection results based on traditional single-level research methods and single-level perspectives deserve further discussion. Therefore, some researchers began to introduce Hierarchical Linear Model (HLM) to analyze the individual effects and background effects of poverty-contributing factors. For example, Cao et al. (2016) took household-level factors as individual effect indicators and village-level factors as background effect indicators to detect the influencing factors of the poverty vulnerability of rural households in Liangshan area of China [25]. Ren et al. (2017) used this model, taking county-level factors as individual effect indicators and district factors as background effect indicators, to detect the influencing factors of poverty incidence at the county level in case study areas of China [26]. Wang et al. (2019) adopted this model, taking village-level factors as individual effect indicators and county-level factors as background effect indicators to detect the poverty-contributing factors in the Wuling destitute area [23]. However, these studies are generally based on two-level detection and paid little attention to three-level one. Most of them only constructed the detection index system of poverty-contributing factors from social capital, economic development, seldom considering the impact of natural conditions such as ecological environment and geographical location on poverty, and seldom analyzed the interaction mechanism of influencing factors among different levels. In addition, restricted by data acquisition, most studies at home and abroad took provincial, municipal, county, village and other geographic unit levels as identifying objects [27–31], and there were fewer studies on multi-level quantitative exploration aiming at micro-level of households. However, taking poor household as identifying unit can not only capture the characteristics of poverty more accurately, but also help local departments provide targeted assistance and household-specific policies [32].

In this context, from the new combined perspectives of multi-level comprehensive detection of poverty-contributing factors and human-nature sustainable development, this study constructs a nested three-level structure of household-level, village-level, and town-level, in which, the village is under the jurisdiction of the town, and is also where farmers live. It will take poor farmer households as the basic research objects, and the meso-micro administrative levels of village and town as the background level, which is more closely related to poor households and may have more direct impacts on their development than other macro-administrative levels. It will aim to construct a multi-level poverty analysis framework based on HLM model that detects the significant poverty-contributing factors of farmers and their interaction mechanism, and tries to answer the following three questions by using an empirical study: (1) Do the poverty-contributing factors have multi-level effects? (2) If so, what are the significant poverty-contributing factors at different levels? (3) How to examine their individual effects, background effects and interactions? By doing so, this study can not only fully detect the impact of factors at all levels on farmers' poverty from multiple dimensions and multiple levels, but also explain the action mechanism of each significant factor on farmers' poverty, further provide technical reference and auxiliary decision support for the precise poverty alleviation.

## Study area and data sources

### Study area

**Fig 1:**
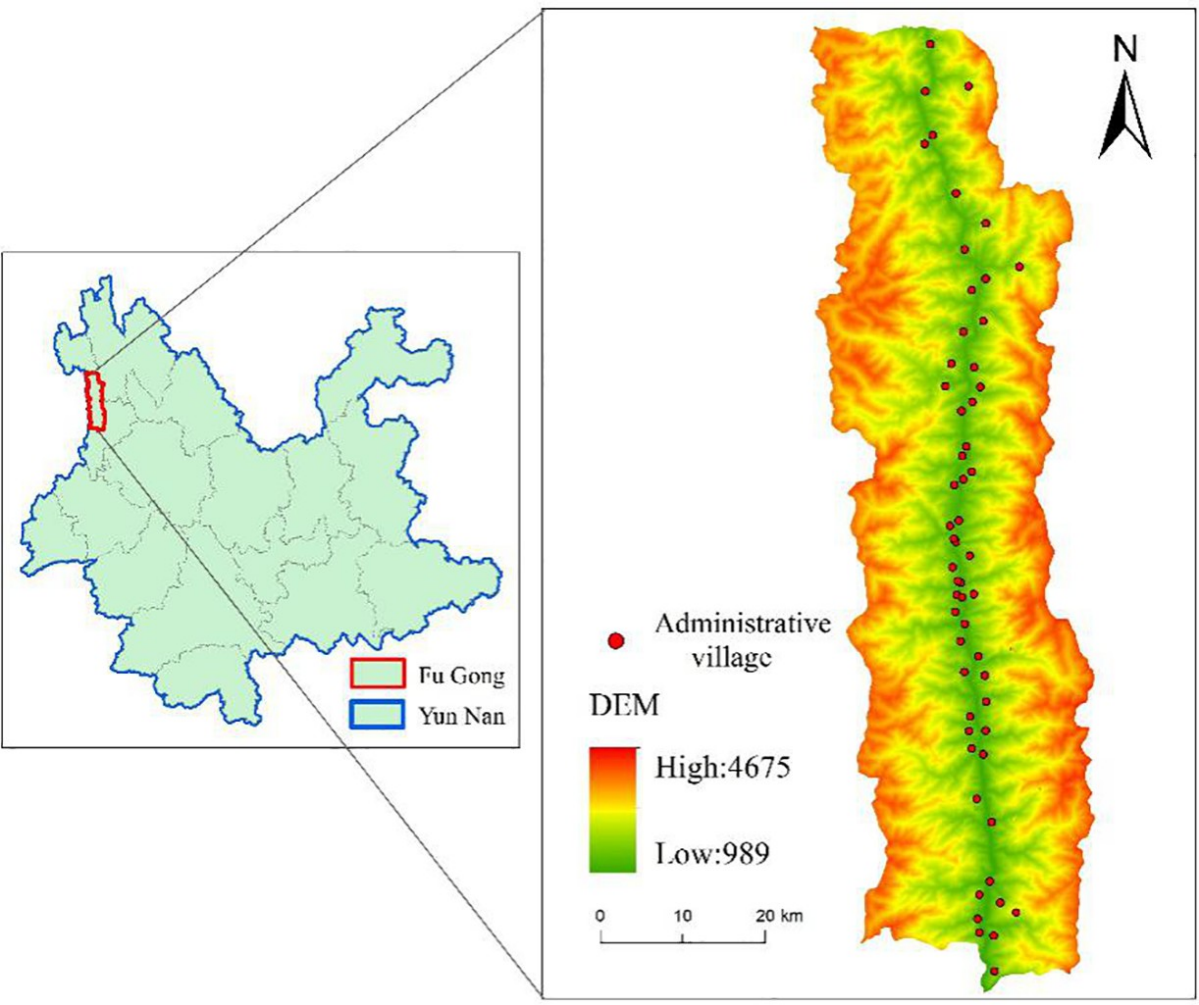
Overview of the study area.

We selected Fugong County in Yunnan Province, China as our case area in this study. Fugong County is one of the key poverty-stricken counties of China, which has a high proportion of poverty, a deep degree of poverty and great difficulty in getting rid of poverty. It acts as the "fortress" for Yunnan Province and even for the whole of China in poverty reduction. As shown in Fig 1, Fugong County belongs to Nujiang Lisu Autonomous Prefecture, located in the Nujiang Gorge between Biluo jokul and Gaoligong Mountain in the middle range of Hengduan Mountains in Northwest Yunnan. It has the high mountains and valley regions, with high north and low south landform. The county covers seven towns, belonging to "the directly-entering-socialism ethnic groups" areas. Five of the seven towns are included in the poverty-stricken towns (except for Magi Town and Xiejia Town), accounting for 72% of the total number of towns. There are 45 poor villages in 57 administrative villages, accounting for 78.95% of the total administrative villages. Thus, the poverty characteristics of Fugong County are very typical, and the poverty problems need to be solved urgently.

### Data sources

Two kinds of socioeconomic and geographic data are used in this study. The socioeconomic data used in this study mainly come from the survey data of poor farmer households, villages, and towns of the study area in 2017. Farmer data include family characteristics, economic status, health, education and so on. Village data include infrastructure, social security, economic development and so on. Town data include social security, economic development and so on. According to the stratified proportional sampling method, the valid samples cover 7 towns, 57 administrative villages and 1205 households in the study area. DEM (Digital elevation model) data with 30m resolution and other geographic data come from Geospatial Data Cloud (http://www.gscloud.cn). All of the above data sets were preprocessed by the joint use of georeferencing, splicing, and clipping, etc.

## Methodology

This study synthetically considers the possible impact of society, economy and geographical environment on poverty, and attempts to blend the farmer livelihood with social exclusion and geographical environment from a multi-level perspective, to design the candidate indicators system of poverty influencing factors at three levels of household, village, and town, and then constructs farmers' poverty-contributing factor detection model based on the hierarchical linear model to systematically detect possible significant poverty-contributing factors and their mechanism among different levels.

### Candidate indicator system for poverty-contributing factors

According to the actual situation of the study area, and following the principles of typicality, representativeness, and independence of the selection of indicators, this paper constructs a three-level indicator system of household-village-town with characteristics of the individual, natural, social and economic situation (Table 1).

**Table 1:** Indicators of household-village-town level.

Level	Type	Variable	Variable interpretation	Coefficient of variation	Complex correlation coefficient
Household	Dependent variable*	*Y_poverty*	Poverty level	—	—
	Geographical location	*F_distance*	Distance from the main road	retain	retain
		*F_road*	Road access type	retain	retain
	Family characteristics	*F_health*	Ratio of healthy family members (%)	retain	retain
		*F_labor*	Ratio of the family labor force (%)	retain	retain
		*F_edu_degree*	Ratio of the population with education below high middle school excepting students (%)	retain	retain
		*F_education*	Ratio of non-compulsory education students in the family (%)	retain	retain
	Social Security	*F_meidcal*	Ratio of the population enrolled in the new rural cooperative medical insurance of China in the family (%)	retain	retain
		*F_insurance*	Ratio of the population enrolled in urban and rural basic pension insurance in the family (%)	retain	retain
	Economic development	*F_income*	Per capita annual income of family (yuan)	retain	retain
Village	Geographical environment	*V_terrain*	Terrain relief	retain	reject
		*V_altitude*	Altitude	retain	retain
		*V_slope*	Slope	retain	retain
		*V_plough*	Per capita cultivated land area	retain	retain
	Infrastructure	*V_road*	Road access ratio (%)	retain	retain
		*V_education*	Education (whether there is a primary school in the village, yes = 1, no = 0)	retain	retain
	Labor situation	*V_labor*	Ratio of village labor force (%)	retain	retain
		*V_worker*	Proportion of migrant workers in the village (%)	retain	retain
	Social Security	*V_medical*	Ratio of the population enrolled in the new rural cooperative medical insurance of China in the village (%)	retain	retain
		*V_pension*	Ratio of the population enrolled in urban and rural basic pension insurance in the village (%)	retain	retain
	Economic development	*V_income*	Per capita annual income of the village (yuan)	retain	retain
		*V_vill_inc*	Collective income of the village (yuan)	retain	retain
Town	Geographical environment	*T_terrain*	Terrain relief	retain	retain
		*T_altitude*	Altitude	retain	retain
	Social Security	*T_hospital*	Number of hospitals in the town	retain	retain
		*T_school*	Number of schools in the town	retain	retain
	Economic development	*T_income*	Per capita annual income of the town (yuan)	retain	retain

Note: The dependent variable is the poverty level (Y), expressed as the per capita income level. According to both the national poverty line and relevant local line of the study area (i.e., national line of poverty standards issued by China’ state council leading group office of poverty alleviation and development in 2011, and local line issued by the poverty alleviation and development department of Yunnan province in 2015), the following grades are divided into 1067 yuan (including 1067 yuan) for absolute poverty, assigned value 5; 1067–2300 yuan for deep poverty, assigned value 4; 2300–2800 yuan for medium Poverty, assigned value 3; 2800–3500 yuan for mild poverty, assigned value 2; more than 3,500 yuan for poverty, assigned value 1. For the entry type indicator, the scoring system is adopted, the asphalt road is assigned value 1, the cement road is assigned value 0.75, the sand road is assigned value 0.5, and the ordinary soil road is assigned value 0.25.

(1) **Household-level.** At the household level, the four dimensions of geographic location, family characteristics, social security, and economic development may have an impact on the poverty level of poor households.

①Geographical location: The distance from the main road and the road access type affect the communication between poor households and the outside world. The geographical location of the occlusion may not be conducive to its development and it is easy to deepen the poverty level.

②Family characteristics: The health status of family members, ratio of the family labor force and ratio of non-compulsory education students in the family show the family medical burden status, labor status and education burden respectively. In addition, the ratio of the population with education below high middle school excepting students (the education of family members) also affects family poverty to a certain extent. Relevant research shows that people with lower education levels have a higher probability of being poor than those with higher education levels [33].

③Social security: The participation rate of new rural cooperative medical insurance and urban and rural basic pension insurance reflects the living burden of farmers and the coverage of national welfare. The heavy burden of family survival and the low national welfare coverage will hinder poor households from getting rid of poverty to a certain extent.

④Economic development: The family economic situation is directly related to the poverty level. In general, the lower the annual income per capita of the family, the deeper the poverty level.

(2) **Village-level.** At the village level, the geographical environment, infrastructure, labor conditions, social security, and economic development may all affect the poverty level of poor households.

①Geographical environment: The quality of the geographical environment affects the living conditions of farmers to a certain extent. Terrain relief, altitude, and slope may have an impact on crop cultivation and traffic conditions. Relevant research shows that the influence of topography and location factors on poverty is increasingly obvious [34] The per capita arable land area reflects the material basis for the survival of farmers.

②Infrastructure: Infrastructure has affected the development of the village to a certain extent. The access rate affects the communication between the villagers and the outside world, and education affects the cultural level of the villagers. Incomplete infrastructure can hinder the advancement of poverty reduction.

③Labor situation: The labor situation affects the income situation to a certain extent. Farmers with good working ability and migrant workers are generally more likely to obtain higher incomes, which will help reduce the poverty level.

④Social security: Social security indicators reflect the coverage of state welfare. New rural cooperative medical care and urban and rural basic pension insurance can help alleviate the burden on families and thus improve the poverty situation.

⑤Economic development: The economic development of the village will affect the development of poor households to a certain extent. According to the trickle effect, economic growth can increase total wealth and promote poverty alleviation [35].

(3) **Town-level.** At the town level, geographical environment, social security, and economic development all have an impact on the poverty level of poor households.

①Geographical environment: The terrain relief and altitude affect the traffic conditions and agricultural production to a certain extent. The highly undulating terrain and high altitude are not conducive to the travel of farmers and the cultivation of agricultural products, thus affecting the communication between farmers and the outside world and the income of farmers.

②Social security: The hospital can help solve the problem of farmers' difficulty in seeing a doctor, and to a certain extent, play a role in preventing major diseases, thereby reducing the incidence of serious illnesses among farmers, thus reducing the risk of the family taking the heavy medical burden. The school provides students with an opportunity to be educated and helps to improve the overall education level. The higher the level of education, the more likely it is to earn a higher income.

③Economic development: The economic development of the town will help to increase the income of farmers' families, thus reducing the level of family poverty.

Based on the above, the household-village-town three-level index system obtained from the subjective primary selection is shown in Table 1. According to the national poverty line standards and the relevant government poverty alleviation policies in the study area, this paper divides the poverty level of poor households into five grades and assigns 1–5 as the dependent variable. The higher the grade, the deeper the poverty level. Further, the variation coefficient method and the complex correlation coefficient method are comprehensively used to select candidate indicators: Firstly, the coefficient of variation of each indicator data at the household, village and town levels is calculated by the coefficient of variation method, and the index with the coefficient of variation greater than 15% (i.e., the data separation degree is larger) is retained. Then, the complex correlation coefficient method is used to carry out the complex correlation simulation on the internal indicators of each dimension of the household, village and town levels, and the indicators with larger complex correlation coefficient are excluded. Since the dimension of the economic development at the town-level has only one indicator, it is only retained according to its coefficient of variation, as shown in Table 1. Finally, 9 household-level indicators, 11 village-level indicators, and 5 town-level indicators are selected for calculation.

### Multi-level poverty-contributing factors detection model

In this paper, the poverty level of farmers is used as the dependent variable, and the candidate index system of poverty-contributing factors constructed above is used as the dependent variable. By constructing household (first level)—village (second level)—town (third level) multi-level farmers' poverty-contributing factor detection system based on HLM model, this paper aims to explore the significant influencing factors of poor households and their interaction mechanism on poor farmers.

Hierarchical Linear Model (HLM) is a statistical method for processing data with nested structure. It can effectively solve the problem of background effect and analyze the degree of influence of independent variables on dependent variables and effect differences at different levels [23, 36]. Because the household-village-town object has a naturally nested structure, that is, the household is embedded in the village and the village is embedded in the town. Therefore, by constructing hierarchical linear models with different combinations, the contribution of the influencing factors to the poverty level can be judged according to the fixed effect (regression coefficient). The random effects (residual terms) are used to judge the difference of contribution degree, and then the mechanism of different poverty-contributing factors is analyzed.

The modeling process of this study is as follows: Firstly, the null model (Model I) is constructed, that is, a model that does not contain any variables, to explore whether there exists a background effect on the poverty level of poor households. Secondly, if the existence of background effect is verified from Model I, it is necessary to construct a random effect model (Model II), that is, a model containing the first level or one or two levels of variables to explore the significant influence factors at the household level and the village level. Finally, a complete model (Model III) is constructed, that is, each level contains a model of variables to explore the significant poverty influencing factors at the town level and the mechanisms at all levels. The calculation of hierarchical linear models in this study is done using HLM 6.08 software, as described below.

#### Model I: Individual effects and background effects detection based on the null model

Before building other models, it is necessary to construct the null model without any indicators to calculate the intra-group differences (house-house differences) of the poverty level, and calculate the differences between the groups (village-village differences and town-town differences) utilizing the ICC index (Intra-Class Correlation Coefficient). According to the size of the background effects at the village level and town level, it is judged whether it is necessary to construct hierarchical models to detect the poverty-contributing factors. Besides, according to the relevant literature [23], when the ICC index is greater than 0.059, it is necessary to add background effects (village-level and town-level factors) to the statistical model to explore the influence of background effects on the poverty level of farmers.

The constructed null model, the intra-group difference and inter-group difference calculation formula are shown in Table 2:

**Table 2:** The calculation formula of the null model and ICC index.

	Expression	Parameter explanation
the null model	Level 1: *Y*_*ijk*_ = *β*_0*jk*_+*r*_*ijk*_ Var(*r*_*ijk*_) = *σ*^2^ Level 2: *β*_0*jk*_ = *γ*_00*k*_+*μ*_0*jk*_ Var(*μ*_*ijk*_) = *τ*_00_ Level 3: *γ*_00*k*_ = *π*_000_+*e*_00*k*_ Var(*e*_*ijk*_) = *τ*_000_	Level 1, Level 2, and Level 3 represent the three levels, household-level, village-level, and town-level. i represents household level units, j represents village-level units, and k represents town-level units. Y_ijk_ represents the poverty level of poor households, β_0jk_ represents the average poverty level of poor households in the village-j of town-k, γ_00k_ represents the average of poverty levels of poor households in K town, π_000_ represents the average of poverty levels of all poor households, r_ijk_, μ_ijk_, e_00k_ is respectively the residual of the first-level, second-level and third-level.
intra-group difference /inter-group difference	intra-group difference: *ρ*_1_ = *σ*^2^/(*τ*_000_+*τ*_00_+*σ*^2^) inter-group difference: *ρ*_2_(*ICC*) = *τ*_00_/(*τ*_000_+*τ*_00_+*σ*^2^) inter-group difference: *ρ*_3_(*ICC*) = *τ*_000_/(*τ*_000_+*τ*_00_+*σ*^2^) *V*ar(*POVERTY*) = *τ*_000_+*τ*_00_+*σ*^2^	σ_2_, τ_00_, τ_000_ respectively represents the variance of the household-level, village-level, and town-level, ρ_1_, ρ_2_, ρ_3_ respectively represents the variance ratio of poverty level at the household-level, village-level, and town-level, that is, the proportion of the impact on the poverty level from the three levels. The equation Var (POVERTY) represents the total variance of the poverty level of the poor households.

#### Model II: Detection of significant poverty-contributing factors at the household-level and the village-level based on random effect regression model

By constructing two random effects regression models, Model II (a) and Model II (b), we explored significant poverty-contributing factors at the household-level and the village-level. In order to more accurately explore the effect of household-level influence factors on poverty level and avoid the influence of the village-level and the town-level factors, Model II(a) only adds explanatory variables at the first level, and substitutes each household-level indicator into the model one by one. Do not add explanatory variables in the second level and the third level in Model II (a). Model II (b) adds the village-level explanatory variables to the second level intercept equation (such as Equation ① in Table 3) to explore the village-level significant factors while adding the household-level influence factors in the first level. In order to more accurately explore the village-level significant poverty-stricken factors and avoid the impact of the town-level background effect on poverty levels, this study do not add explanatory variables in the third level in Model II (b). The two three-level random effects regression models constructed are shown in Table 3:

**Table 3:** Random effect regression model.

	Model expression	Parameter explanation
Model Ⅱ(a)	Level 1: *Y*_*ijk*_ = *β*_0*jk*_+*β*_1*jk*_*X*_1*ijk*_+*r*_*ijk*_ Level 2: *β*_0*jk*_ = *γ*_00*k*_+*μ*_0*jk*_ *β*_1*jk*_ = *γ*_10*k*_+*μ*_1*jk*_ Level 3: *γ*_00*k*_ = *π*_000_+e_00*k*_ *γ*_10*k*_ = *π*_100_+e_10k_	X_1ijk_ is the explanatory variable of the household-level, and β_1jk_ is its regression coefficient (the contribution of the household-level factor to the poverty level). γ_10k_ is the average value of β_1jk_ in town-k. π_100_ represents the overall average value of β_1jk_. μ_1jk_ and e_10k_ are respectively the residual of β_1jk_ and γ_10k_.
Model Ⅱ(b)	Level 1: *Y*_*ijk*_ = *β*_0*jk*_+*β*_1*jk*_*X*_1*ijk*_+*r*_*ijk*_ Level 2: *β*_0*jk*_ = *γ*_00*k*_+ *γ*_01*k*_*W*_1*jk*_+*μ*_0*jk*_^①^ *β*_1*jk*_ = *γ*_10*k*_+*μ*_1*jk*_ Level 3: *γ*_00*k*_ = *π*_000_+e_00*k*_ *γ*_01*k*_ = *π*_010_+e_01*k*_ *γ*_10*k*_ = *π*_100_+e_10*k*_	W_1jk_ is the explanatory variable of the village-level, γ_01k_ (the contribution of village-level factors to poverty level) is the regression coefficient of W_1jk_ that is related to β_0jk_. π_010_ is the average value of γ_01k_. e_01k_ is the residual of γ_01k_. The rest of the variables are explained in the same way as model II(a).

#### Model III: Significant poverty-contributing factors and the interaction mechanism at household-village-town levels based on the full model

In this stage, we construct model III (a) based on model II (b). We add the town-level influencing factors in the intercept equation into the third level (as in Equation ② in Table 4), and add residuals to each equation of the model to explore the significant influence factors at the town-level, as well as the difference of the contribution of the household-level factors and the village-level factors to the poverty level. Further, based on model III (a), the village-level and the town-level explanatory variables are added to the regression coefficient equations of the household-level and village-level influencing factors with significant differences in contribution to construct model III (b), which explains the possible reasons for the significant difference of contribution. The three-level complete model constructed is shown in Table 4:

**Table 4:** The full model.

	Model expression	Parameter explanation
Model Ⅲ(a)	Level 1: *Y*_*ijk*_ = *β*_0*jk*_+*β*_1*jk*_*X*_1*ijk*_+*r*_*ijk*_ Level 2: *β*_0*jk*_ = *γ*_00*k*_+ *γ*_01*k*_*W*_1*jk*_+*μ*_0*jk*_ *β*_1*jk*_ = *γ*_10*k*_+*μ*_1*jk*_ Level 3: *γ*_00*k*_ = *π*_000_+*π*_001_*Z*_00*k*_+e_00*k*_^②^ *γ*_01*k*_ = *π*_010_+e_01*k*_ *γ*_10*k*_ = *π*_100_+*e*_10*k*_	Z_00k_ is the explanatory variable of the town-level, and π_001_ (the contribution of town-level factors to poverty level) is its regression coefficient. The rest of the variables are explained in the same way as model II(a).
Model Ⅲ(b)	Level 1: *Y*_*ijk*_ = *β*_0*jk*_+*β*_1*jk*_*X*_1*ijk*_+*r*_*ijk*_ Level 2: *β*_0*jk*_ = *γ*_00*k*_+ *γ*_01*k*_*W*_1*jk*_+*μ*_0*jk*_ *β*_1*jk*_ = *γ*_10*k*_+ *γ*_11*k*_*W*_1*jk*_+*μ*_1*jk*_ Level 3: *γ*_00*k*_ = *π*_000_+*π*_001_*Z*_00*k*_+e_00*k*_ *γ*_01*k*_ = *π*_010_+*π*_011_*Z*_01*k*_+e_01*k*_ *γ*_10*k*_ = *π*_100_+*π*_101_*Z*_10*k*_+e_10*k*_ *γ*_11*k*_ = *π*_110_+*π*_111_*Z*_11*k*_+e_11*k*_	W_1jk_ is the explanatory variable of the village. γ_11k_ (the contribution of village-level factors to poverty level) is the regression coefficient of W_1jk_ that is related to β_1jk_, and it is used to explain the significant difference of the contribution (β_1jk_) among household factors to poverty. π_110_ is the average value of γ_11k._ Z_01k_, Z_10k_, and Z_11k_ are respectively the explanatory variable of γ_01k_, γ_10k_, γ_11k_. e_01k_, e_10k_, e_11k_ are respectively the residual of γ_01k_, γ_10k_, γ_11k_. The rest of the variables are explained in the same way as model II(a).

## Results and analysis

### Individual effect and background effect of poverty-contributing factors

The calculation results of the null model are shown in Table 5:

**Table 5:** Calculation results of the null model.

Fixed effect				Random effect			
Parameter	Regression coefficients	Standard deviation	T value	Parameter	Variance component	Chi-square value	Variance ratio
G000 (*β*_*0jk*_)	2.634	0.197	13.406***	*E* (*σ*^*2*^)	1.160	—	0.7714 (*ρ*_*1*_)
				*R0* (*τ*_*0*_)	0.094	132.96802***	0.0624 (*ρ*_*2*_)
				*U00* (*τ*_*00*_)	0.250	91.40612***	0.1662 (*ρ*_*3*_)

Note

* p< 0.1

** p <0.05

*** p<0.01.

The estimated results of the null model (Table 5) show that both the fixed effect and random effect examination reached a significant level. The correlation coefficients of intra-group (household-household) and inter-group (village-village, town-town) of household-village-town levels calculated by the ICC index are 0.7714, 0.0624 and 0.1662, respectively, which are greater than 0.059. In other words, 77.14% of the variation among the households’ poverty degrees comes from the development variations among poverty-stricken households, 6.24% comes from variations among villages they belong to, and 16.62% comes from variations among towns they belong to. And the background effect on village-level and town-level need to be added to the statistical model. That is to say, a multi-level linear regression model is needed to detect the poverty-contributing factors of farmers.

### Multi-level poverty-contributing factors

Based on the estimation results of model II and model III, Table 6 shows that the contribution of poverty-contributing factors to the households’ poverty degrees at household-level, village-level, and town-level. This paper will analyze the significant poverty-contributing factors at the three levels of household, village, and town.

**Table 6:** Factors affecting the poverty level of poor households.

Household-level			Village-level			Town-level		
Explanatory variables	Intercept (*β*_*0jk*_)	Regression coefficients (*β*_*1jk*_)	Explanatory	Parameter	Regression coefficients (*γ*_*01k*_)	Explanatory	Parameter	Regression coefficients (*π*_*001*_)
*F_distance*	2.635***	0.036	*INTRCPT*	*G000*	*2*.619***	*INTRCPT*	*G000*	*2*.*620*
*F_road*	2.637***	-0.108**	*V_altitude*	*G010*	*0*.*016*	*T_terrain*	*G001*	*0*.331***
*F_health*	2.634***	-0.009	*V_slope*	*G020*	*0*.481**	*T_altitude*	*G002*	*0*.*115*
*F_labor*	2.634***	-0.144***	*V_plough*	*G030*	*-3*.309**	*T_hospital*	*G003*	*0*.*065*
*F_edu_degree*	2.635***	0.042**	*V_road*	*G040*	*3*.082**	*T_school*	*G004*	*-0*.*152*
*F_education*	2.636***	-0.074	*V_education*	*G050*	*-0*.*038*	*T_income*	*G005*	*-0*.313**
*F_meidcal*	2.634***	0.068***	*V_labor*	*G060*	*-0*.*070*			
*F_insurance*	2.633***	-0.122**	*V_worker*	*G070*	*-0*.104**			
*F_income*	2.615***	-1.629***	*V_medical*	*G080*	*-0*.*042*			
			*V_pension*	*G090*	*-0*.*057*			
			*V_income*	*G0100*	*-0*.*024*			
			*V_vill_inc*	*G0110*	*0*.*007*			

Note

* p< 0.1

** p <0.05

*** p<0.01.

(1) **Household-level.** According to the absolute value of regression coefficients of each model, the contribution of significant poverty-contributing factors from high to low are per capita annual income of family (-1.629), ratio of family labor force (-0.144), ratio of population enrolled in urban and rural basic pension insurance (-0.122), road access type (-0.108), ratio of population enrolled in the new rural cooperative medical insurance of China (0.068) and the ratio of population with education below high middle school excepting students (0.042). ①There is a significantly negative correlation between the per capita annual income of the family and poverty degree. In other words, the lower the per capita annual income of the family is, the higher the poverty degree is. ②There is a significant negative correlation between the proportion of household labor force and poverty degree. That is to say, the lower the ratio of the family labor force is, the higher the poverty degree is. The family labor force affects family poverty degree by influencing family income. ③ Ratio of the population enrolled in urban and rural basic pension insurance has a significantly negative correlation with poverty degree. That is to say, the higher the ratio of the population enrolled in urban and rural basic pension insurance is, the lower the poverty degree is. Pension insurance will reduce the burden of family support for the old, and alleviate family poverty degree. ④Road access type has a significantly negative correlation with poverty degree. That is to say, the worse road access type is, the higher poverty degree is. The reason is that the inconvenience of transportation will hinder communication between farmers and the outside world as well as development. ⑤Ratio of the population enrolled in the new rural cooperative medical insurance is positively correlated with the degree of poverty. That is to say, the higher the ratio of the population enrolled in the new rural cooperative medical insurance is, the higher the poverty degree is. This reflects that the poorer the family's ability to resist the risk of disease, the higher the poverty degree. ⑥There is a significant positive correlation between the ratio of the population with education below high middle school excepting students and poverty degree. That is to say, the more people with low or middle school education is, the higher the poverty degree is. People possibly have the low income level who has the low education level, which is not conducive to alleviating family poverty.

(2) **Village-level.** According to the absolute value of regression coefficients of each model, the significant poverty-contributing factors from high to low are per capita cultivated land area (-3.309), road access ratio (3.082), slope (0.481) and the proportion of migrant workers (-0.104). ①The per capita cultivated land area is negatively correlated with poverty degree. That is to say, the less arable land area per capita is, the higher the poverty degree is. Farmland provides the material conditions for families to survive and directly affects family income, thus affecting family poverty degree. ②There is a significant positive correlation between road access ratio and poverty degree. That is to say, the higher the road access ratio is, the higher the poverty degree is. Due to the special topography and geomorphology of the study area, the rural households are scattered and many roads are “stepped out”. The road number indicates that the farmers are inconvenient to communicate, and the poverty alleviation work is also inconvenient to be carried out because the farmers are not well dispersed, which is not conducive to the poverty reduction of farmers. ③There is a significant positive correlation between the slope and poverty degree. That is to say, the greater the slope is, the higher the poverty degree is. The larger slope will bring inconvenience to villagers in transportation, crop cultivation, and other aspects, which is not conducive to the economic development of farmers. ④There is a significant negative correlation between the proportion of migrant workers in villages and the poverty degree. That is to say, the more migrant workers there are, the lower the poverty degree is. Migrant workers are less affected by natural factors than farming, and their income is more stable, which is conducive to reducing family burden and poverty.

(3) **Town-level.** According to the absolute value of regression coefficients of each model, the significant poverty-contributing factors from high to low are terrain relief (0.331) and per capita annual income (-0.313). ①There is a significantly positive correlation between terrain relief and poverty degree. That is to say, the higher the terrain relief is, the higher poverty degree is. Because the larger topographic fluctuation is not conducive to crop cultivation. To a certain extent, it will reduce the cultivated area, thus affecting the supply of material living conditions of farmers and the poverty situation of farmers. ②The annual per capita net income of town peasants is negatively correlated with poverty degree. That is to say, the lower annual per capita net income of town peasants is, the higher poverty degree is. As individual economic development will be affected by the collective economy, the economic development of town will have an impact on the economic development of farmers. The good development of the town is conducive to the improvement of farmers' income and the reduction of poverty.

### Interaction mechanism of multi-level poverty-contributing factors

By comparing random effects of the null model and model III (a) (Table 7), it can be concluded that the significant influencing factors at the household level contribute 70.95% of poverty gap at the household level, 11.70% of the difference in the poverty level at the village level and 86.80% of the difference in the poverty level at the town level.

**Table 7:** The comparison of random effects of the null model and Model III (a).

the null model			Model Ⅲ(a)		
Parameter	Variance	Variance ratio	Parameter	Variance	Variance ratio
*E* (*σ*^*2*^)	1.160	0.7714	*E* (*σ*^*2*^)	0.337	70.95%
*R0* (*τ*_*0*_)	0.094	0.0624	*R0* (*τ*_*0*_)	0.083	11.70%
*U00* (*τ*_*00*_)	0.250	0.1662	*U00* (*τ*_*00*_)	0.033	86.80%

From random effect of model III (a) (Table 8), it can be concluded that there are significant differences among villages in the contribution of three indicators, which are ratio of population enrolled in the new rural cooperative medical insurance of China, ratio of population enrolled in urban and rural basic pension insurance and per capita annual income of family to poverty degree. There are significant differences among towns in the contribution of the proportion of migrant workers to the poverty degree. And the contribution of per capita annual income of family to poverty degree is significantly different among towns. Therefore, it is necessary to add variables at the village level and the town level to explore the poverty gap of household and village factors.

**Table 8:** The comparison of random effects of Model III (a) and Model III (a).

Model Ⅲ(a)				Model Ⅲ(b)				Village level	Town level
Parameter	Variance (*τ*_*0*_)	Parameter	Variance (*τ*_*00*_)	Parameter	Variance (*τ*_*0*_)	Parameter	Variance (*τ*_*00*_)	Variance ratio	Variance ratio
*R0*	*0*.*083*	U00	0.033***	*R0*	0.095***	U00	0.040***		
*R1*	*0*.*001*	*U01*	*0*.*017*	*R1*	*—*	*U01*	*—*		
*R2*	*0*.*005*	*U02*	*2*.*943*	*R2*	*—*	*U02*	*—*		
*R3*	*0*.*004*	*U03*	*2*.*881*	*R3*	*—*	*U03*	*—*		
*R4*	*0*.004**	*U04*	*0*.032*	*R4*	0.003*	*U04*	0.013	25%	59.38%
*R5*	*0*.005***	*U10*	*0*.*000*	*R5*	0.002	*U10*	*—*	*60%*	
*R6*	*0*.618*	*U20*	*0*.*002*	*R6*	0.553***	*U20*	*—*	*10%*	
*E* (*σ*^*2*^)	*0*.*337*	*U30*	*0*.*000*	*E* (*σ*^*2*^)	0.348	*U30*	*—*		
		*U40*	*0*.*001*			*U40*	*—*		
		*U50*	*0*.*003*			*U50*	*—*		
		*U60*	*0*.089*			*U60*	0.009		89.89%

Note

* p< 0.1

** p <0.05

*** p<0.01.

From the variance significance and comparison results of Table 8, it can be found that the contribution of ratio of population enrolled in the new rural cooperative medical insurance of China to the poverty level of farmers is different among different villages and 25% [(0.004–0.003)/0.004] of the difference (*R4*) is related to the medical care, insurance coverage, per capita income and collective income of each village. The contribution of the ratio of the population enrolled in urban and rural basic pension insurance to the poverty level of farmers is different among different villages and 60% [(0.005–0.002)/0.005] of the difference (*R5*) is related to the above four village factors. The contribution of per capita annual income of family to the poverty level of farmers is different among different villages and 10% [(0.618–0.553)/0.618] of the difference (*R6*) is related to per capita annual income of each village as well as collective income of the village. The contribution of the proportion of migrant workers at the village level to the poverty level of farmers is different in different towns and 59.38% [(0.032–0.013)/0.032] of the difference (*U04*) is related to terrain relief at the town level and the per capita annual income of town. The contribution of per capita annual income of family to the poverty level of farmers is different among different towns and 89.89% [(0.089–0.009)/0.089] of the difference (*U60*) is related to the above two town-level factors.

In short, the four factors of village-level medical treatment, insurance coverage and village per capita annual income and collective income have influence on the difference of poverty degree of farmers caused by household-level factors including ratio of population enrolled in the new rural cooperative medical insurance of China, ratio of population enrolled in urban and rural basic pension insurance and per capita annual income of family. Terrain relief and per capita annual income at the town level has an influence on the difference of poverty degree of farmers caused by the village-level factor (proportion of migrant workers) and household-level factor (per capita annual income of family).

## Conclusions and discussions

The previous researches on poverty-contributing factors of farmer households mostly stay at a single level (provincial or municipal level) and seldom consider the comprehensive interaction effects of social, economic and ecological factors among multiple levels. To respond to it, this paper designs a detection model of poverty-contributing factors of farmers considering multi-level individual effect and background effect from the three levels of household-level, village-level and town-level, revealing the significant poverty-contributing factors and their interaction mechanism. The empirical results from the study area show that: (1) Poverty- contributing factors are multi-level, showing both individual and background effects. Farmers' poverty is not only affected by individual effect at the household level, but also by background effect at the village level and town level. Specifically, 77.14% of the poverty is caused by household-level factors, 6.24% by village-level factors and 16.62% by town-level factors. (2) Significant poverty-contributing factors at different levels are different, identifying different contribution degrees to poverty gaps of farmer households. Five household-level factors show significant influence on poverty degree and account for 70.95% of the overall poverty gap among poor households, which are road access type, ratio of family labor force, educational level of family members, ratio of population enrolled in the new rural cooperative medical insurance, and ratio of population enrolled in urban and rural basic pension insurance, respectively. At the village level, four factors, i.e., slope, per capita cultivated land area, road access ratio and proportion of migrant workers, have significant effects on the poverty degree, and account for 11.70% of the overall poverty gap of poor households. At the town level, terrain relief and per capita annual income has significant impacts on the poverty level of poor households and contribute 86.80% of the overall poverty gap of poor households. (3) Higher-level factors have different degrees of influence on the contribution difference of lower-level factors. Four village-level factors, i.e., medical care, insurance coverage, per capita annual income and collective income, have explained 25% of the difference of ratio of population enrolled in the new rural cooperative medical insurance's contribution to poverty degree among villages and 60% of the difference of ratio of population enrolled in urban and rural basic pension insurance’s contribution to poverty degree among villages, respectively. The two factors of village-level factors, per capita annual income and collective income, have explained 10% of the difference of household-level per capita annual income’s contribution to poverty degree among villages. The two town-level factors of terrain relief and town per capita annual income have explained 59.38% of the difference of village-level proportion of migrant workers’ contribution to poverty degree among towns and 89.89% of the difference of household-level per capita annual income's contribution to poverty degree among towns respectively.

According to the survey statistics and the results of the above experiments in the study area, combined with the requirements of the national precise poverty alleviation policy of China and the actual situation of the study area, measures such as improving type of access roads, developing characteristic planting and breeding and implementing relocation projects can help poor households in the study area to reduce poverty. The following suggestions are given to provide a reference for the poverty reduction in the study area. Firstly, there are some suggestions at the household level. (1) Road access type needs to be improved, which can facilitate farmers to travel and strengthen their contacts with the outside world. (2) The coverage of national welfare, such as medical care and insurance needs to be increased, which can reduce the possibility of poverty among farmers. (3) Poverty alleviation should firstly improve "wisdom" because the low level of education of farmers will largely restrict their own development. In addition, due to the low level of education, it may be difficult to understand and coordinate the national policy, which will hinder the development of poverty alleviation work. Secondly, there are some suggestions at the village level. (1) Characteristic planting and aquaculture should be further developed. Because of the special terrain of villages in the study area, it is very difficult to realize the traditional large-level farming, and the harvest is relatively not optimistic. Therefore, it is necessary to develop new planting and aquaculture industries to improve the farmer's income. (2) Local government can implement poverty alleviation through rural tourism and build tourism demonstration households. Rural tourism can not only increase the income of local farmers but also reduce the proportion of migrant workers to a certain extent, thus improving the situation of left-behind children and empty-nest elderly. (3) Roads need to be further built, which can increase the accessibility of natural villages, ensure road safety in poor villages, facilitate exchanges between villages and the outside world, and promote the development of villages. Finally, there are some suggestions at the town level. (1) Implementing the relocation project through changing habitat is beneficial to poverty alleviation. Due to the influence of terrain relief, the distribution of villages in the town is scattered, which is not conducive to the management and poverty alleviation. Setting up centralized resettlement sites and improving infrastructure can improve the living environment and farmers' well-being. (2) The health poverty alleviation project needs to be improved. Related departments should build county hospitals, town hospitals, and village clinics, strengthen the training of medical technicians, improve the professional level of doctors and prevent endemic and key diseases in time to reduce the possibility of poverty caused by diseases. (3) The construction of the talent team needs to be strengthened. Training and selecting grass-roots cadres such as college students and village officials can lead the economic and social development of the research area. Training professionals and technical talents can promote the development of innovative industries in the research area. Introducing special post teachers and master of education in rural areas promotes the development of local education. And allocating free medical students to predetermined county and town medical and health institutions improves the overall professional quality and medical level of doctors.

In addition, this study does have some limitations. For example, the distribution of spatial poverty and the change of poverty-contributing factors in time series are not taken into account in this paper. Therefore, more attention should be paid to the changes in poverty-contributing factors and their spatial level effects based on panel data in the future to provide more targeted policy reference for precise poverty alleviation.
